# A medical assistant-facilitated transition activity in a pediatric cardiology clinic

**DOI:** 10.1016/j.hctj.2024.100042

**Published:** 2024-01-18

**Authors:** Debora Burger, Quin E. Denfeld, Karen Uzark, Patrick D. Evers, Andrew W. McHill, Pam Ward, Reem Hasan

**Affiliations:** a Oregon Health & Science University, School of Nursing, Portland, OR, USA; b Oregon Health & Science University, Knight Cardiovascular Institute, Portland, OR, USA; c University of Michigan Mott Children’s Hospital, Division of Pediatric Cardiology, Ann Arbor, MI, USA; d Oregon Health & Science University, Division of Pediatric Cardiology, Portland, OR, USA; e Oregon Health & Science University, Departments of Internal Medicine and Pediatrics, Portland, OR, USA

**Keywords:** Transition, Congenital heart disease (CHD), Medical assistant (MA)

## Abstract

**Background:**

Formal transition programs prepare pediatric patients with congenital heart disease (CHD) for successful lifelong management of their disease. Conducting transition program activities in pediatric cardiology clinics can be a challenge if there are limited resources. The purpose of this study was to test the effectiveness of a medical assistant (MA)-facilitated transition activity in increasing documentation of transition discussions and characterize staff acceptability of this intervention.

**Method:**

We performed a prospective exploratory study over a five-week period. CHD patients aged 13 and older presenting for routine pediatric cardiology follow-up appointments received a prompt from the MA to view a list of 17 transition topics from which to choose topics for discussion with the pediatric cardiologist during the clinic visit. Historical control group data were collected from the same period, two years prior. We compared the presence of documentation of transition discussions between the transition activity and control group using comparative statistics. Staff acceptability was assessed using the revised Treatment Acceptability and Preference Questionnaire.

**Results:**

A total of 14 staff members participated in the transition activity involving 29 patients. Significantly more transition discussions were documented in the transition activity group compared with the historic control group (*p* < 0.001). Patients discussed more transition topics (median = 5, Interquartile range 2–7) than what was requested (median = 2, Interquartile range 1–4). All staff rated the activity as acceptable (ranging from ‘somewhat acceptable’ to ‘very much acceptable’) and were willing to continue after the study ended.

**Conclusion:**

Having an MA-facilitated transition activity increased documentation of transition discussions in the pediatric cardiology clinic. Staff were accepting and in favor of continuing this low-resource activity.

## Highlights

• Conducting transition program activities in pediatric cardiology clinics can be a challenge if there are limited resources.
• We tested a Medical Assistant-facilitated transition activity in this setting.
• The activity increased documentation of transition discussions.
• Staff were accepting and in favor of continuing this activity.

## 1 Introduction

Formal transition programs aim at preparing pediatric patients with congenital heart disease (CHD) for successful lifelong management of their chronic disease by fostering knowledge of their CHD and self-management and self-advocacy skills.^1^, ^2^ These programs are important because many teenagers lack knowledge about their condition and are unprepared for transfer to adult care,^3^ and a lack of transition education is associated with decreased self-efficacy and self-management skills.^4^ Additionally, increased patient preparation may alleviate anxiety and uncertainty surrounding the transfer process to adult care.^5^ Moreover, pediatric patients with CHD are also interested in learning about transition topics and understanding their heart defects and cardiac surgeries.^6^ Formal transition programs have demonstrated improvements in adherence to care, improved quality of life, improved patient self-care, improved satisfaction with the health care team, and improved healthcare utilization.^7^ This preparation is best if started between 12–14 years of age.^1^

Even though we know that transition programs are effective, how best to deliver these programs remains undetermined.^1^ Conducting transition program activities in pediatric cardiology clinics can be a challenge if there is limited clinic space, time, and staff to perform these activities.^1^ Expanded roles for medical assistants (MAs) have been explored in the primary care setting with intentions to decrease physician workload and improve patient care.^8^ For example, MAs serving as health coaches resulted in improved diabetes and lipid management in low-income patients.^9^ In pediatric cardiology, MAs may be able to facilitate transition discussions by introducing a transition tool that prompts discussions between the patient and the healthcare team. By facilitating transition discussions, MAs may alleviate the need for additional resources to perform transition activities. The purpose of this study was to test the effectiveness of increasing discussion about transition and characterize staff acceptability of a MA-facilitated transition activity in the ambulatory care setting.

## 2 Methods

### 2.1 Study design

We performed a prospective exploratory study of a MA-facilitated transition activity in a pediatric cardiology clinic located in a tertiary medical center. We conducted this work over a five-week period from June to July 2023. We selected this time frame as transition-aged patients are typically out of school on summer vacation and more likely to attend the pediatric cardiology clinic. Control group data were collected by retrospective review over a five-week period from the same time period as the study but two years prior. We selected June/July 2021 as the comparison group for transition documentation as this was before we embarked on a hospital-wide effort to improve transition service delivery. The pediatric cardiology clinic had resumed routine clinic operations, previously impacted by the COVID-19 pandemic, prior to the historical group data collection. Transition discussions for the control group took place at the discretion of the pediatric cardiologist and they may or may not have been documented in the medical record. At the end of the study, staff completed a questionnaire about their acceptance of the transition activity. The study was approved by the Oregon Health & Science University Institutional Review Board. A consent information sheet was attached to the staff questionnaire and completion of the questionnaire implied informed consent.

### 2.2 Sample

Our target staff population was comprised of MAs (n = 2), pediatric cardiologists (n = 6), pediatric cardiology fellows (n = 2), advanced practice providers (n = 2) and pediatric cardiology nurses (n = 2) who provided care for patients eligible for transition per standard clinical practices and met the following criteria: English-speaking, age 13 and older, and presenting for routine pediatric cardiology follow-up appointments for CHD. Patients with a history of heart transplants were also included in the study. Patients with documentation of intellectual disability on their problem list in the medical record were excluded from the study, unless there was documentation in the last pediatric cardiology clinic note that the intellectual disability was mild. We chose this approach as patients with intellectual disability have an established transition pathway in our clinic that includes an individualized assessment of learning needs. The required qualifications and duties of an MA vary nationally and internationally. The usual duties of the MA in our study are rooming, collecting patient vital signs, point of care testing, electrocardiograms, documentation in the electronic medical record, maintaining clinic flow, stocking exam rooms, and assisting with procedures and treatments. Detailed characteristics of the staff sample were not obtained to protect the identity of the participants.

### 2.3 Procedures

The study was presented by one member of the research team at a pediatric cardiology division meeting. MAs and pediatric cardiology nurses also attend the division meetings. Those who were unable to attend the division meeting received individual education. A script was developed for the MA team to use when presenting the study to patients. A standard electronic health record phrase was developed for the MA team to record the patient and provider transition discussion in the electronic medical record. Using scripted prompts, MAs instructed patients to review a transition handout containing 17 transition related topics. The handout was developed by Uzark and colleagues (2015) to guide transition discussion between the patient, family member(s), and the pediatric cardiologist.^4^ The MA prompted patients to circle the transition topic(s) of interest during routine clinic rooming activities. The handout was then given to the pediatric cardiologist. The pediatric cardiologist indicated which transition topics were discussed during the visit and the completed transition encounter was documented in the clinic note by the MA. The historical control group received usual MA activities only.

### 2.4 Data collection

We collected data of documentation of transition discussions during the clinic visit. Control group (i.e., historical) data was collected from a retrospective medical record review for the presence of documentation of transition discussions in the office visit note. We collected baseline patient data including age, sex and primary cardiac diagnosis. We then grouped patients’ cardiac anatomy by the 2018 American Heart Association (AHA) / American College of Cardiology (ACC) guideline for the management of adults with CHD ACHD Anatomic and Physiological (AP) classification system as simple, moderate complexity and great complexity CHD.^10^ Patients with a history of heart transplants were grouped separately.

### 2.5 Outcomes

Staff outcomes included staff acceptance of the transition activity. Perceived staff acceptability of the activity was measured by the acceptability portion of the revised Treatment Acceptability and Preference Questionnaire, which has demonstrated reliability and validity.^11^ The measure of acceptability captures perceived acceptability of an intervention. It includes four subscales: effectiveness (i.e., does the activity achieve its goal), appropriateness (i.e., is the activity logical and reasonable), suitability (i.e., for the clinic setting) and willingness to comply.^11^ The questionnaire also contains an additional measure of risk (i.e., did the activity produce any adverse effects). Each of the subscales are rated on a five-point scale, ranging from not at all (0) to very much (4). Scores were computed for each subscale and for the overall acceptability of the activity, with higher scores indicating more acceptability. The questionnaire contained an additional open-ended question: is there anything else you want to tell us? Questionnaires were emailed to staff members at the end of the study using the Qualtrics platform (Qualtrics, Provo, UT).

### 2.6 Data analysis

Standard descriptive statistics of frequency, central tendency, and dispersion were used to describe the findings. Independent t-tests were used to test the difference between the number of transition topics documented between the transition activity group and the historical control group. Independent t-tests were also used to test the differences between the number of transition discussions that patients in the transition activity group requested and the number that were addressed during the clinic visit. Individual and a total scale score retrieved from the completed revised Treatment Acceptability and Preference Questionnaire were computed as the mean of the items’ scores to reflect level of perceived treatment acceptability. SPSS version 28 (IBM, Armonk, NY) was used to perform data analyses.

## 3 Results

A total of 29 patients received the MA-facilitated transition activity as part of this pilot study. The mean age of patients receiving the activity was 16.3 ± 1.8 years, ranging between 13- 20 years of age. Half of the sample (52%) were male. In an examination of CHD anatomy, one patient had simple complexity, 18 patients had moderate complexity, and eight patients had great complexity CHD anatomy. Two patients had undergone heart transplantation. A total of 24 patients were eligible for the retrospective control group.

### 3.1 Transition discussions

There were significant differences in the total number of documented transition topics between the transition activity group and the retrospective control group (143 topics discussed across 29 patient visits vs 1 topic discussed across 24 patient visits, respectively, *p* < 0.001). Notably, the transition activity group discussed significantly more transition topics (median 5 [interquartile range 2–7]) than what was requested on the transition handout (median 2 [interquartile range 1–4]; *p* < 0.001). ‘The name of your heart condition and any surgeries’ (11, 38%) and ‘The names of your medications and what they are used for’ (10, 35%) were the top two requested and discussed topics. All transition topics except one (‘How to talk to friends and others about your heart’) were discussed among more patients than what was originally requested. (Table 1).

**Table 1:** Transition Topics Requested and Discussed, n = 29.

**Transition activity group**	**Requested**	**Discussed**
The name of your heart condition and any surgeries	11 (37.9%)	17 (58.6%)
The names of your medications and what they are used for	10 (34.5%)	17 (58.6%)
Symptoms or problems doctor needs to know about	8 (27.6%)	11 (37.9%)
How to talk with your doctor and ask questions	0	8 (27.6%)
How to contact your heart doctor or nurse	7 (24.1%)	13 (44.8%)
If you need antibiotics for dental work	3 (10.3%)	6 (20.7%)
How to take your medications correctly without help	1 (3.4%)	2 (6.9%)
Exercise or sports recommendations	6 (20.7%)	8 (27.6%)
How to talk to friends and others about your heart	3 (10.3%)	3 (10.3%)
Future needs for cardiology visits	10 (34.5%)	11 (37.9%)
How pregnancy might affect your heart and your baby	3 (10.3%)	5 (17.2%)
How to prevent pregnancy with the safest birth control	2 (6.9%)	3 (10.3%)
How to refill your prescriptions	5 (17.2%)	8 (27.6%)
How to make your appointments	7 (24.1%)	11 (37.9%)
How to manage your stress	4 (13.8%)	5 (17.2%)
Job or vocational counseling	3 (10.3%)	5 (17.2%)
Health insurance needs when not covered by your parents	8 (27.6%)	10 (34.5%)

### 3.2 Acceptability

Of the 14 staff members who participated in the study, a total of 11 (79%) completed the acceptability portion of the revised Treatment Acceptability and Preference Questionnaire^12^ (Table 2). The acceptability of the activity was rated by all staff members as somewhat acceptable to very much acceptable (score mean 3.23 ± 0.3). Medical assistants ranked all questions as ‘very much acceptable.’ All staff ranked the activity as ‘not at all’ for risk. Staff responses demonstrated a willingness to utilize the activity on an ongoing basis. ‘How effective do you think this activity is in preparing teenagers/ young adults with congenital heart disease for transfer to adult congenital heart disease care?’ and ‘How effective do you think this activity is in improving your ability to perform your daily usual clinic activities?’ were ranked lowest by staff.

**Table 2:** Staff Acceptability of the Transition Activity.

	**0**	**1**	**2**	**3**	**4**	**Items ranking 3 and 4**	**Median (IQR)**
**Effectiveness**	Not effective at all	Somewhat effective	Effective	Very effective	Very much effective		
How effective, in the short-term, do you think this activity is in increasing transition discussions with teenagers/ young adults with congenital heart disease in the pediatric cardiology ambulatory clinic setting?	0	0	0	7 (63.6%)	4 (36.4%)	11 (100%)	3 (3-4)
How effective, in the long-term, do you think this activity will be in increasing transition discussions with teenagers/ young adults with congenital heart disease in the pediatric cardiology ambulatory clinic setting?	0	0	0	6 (54.5%)	5 (45.5%)	11 (100%)	3 (3-4)
How effective do you think this activity is in preparing teenagers/ young adults with congenital heart disease for transfer to adult congenital heart disease care?	0	1 (9.1%)	3 (27.3%)	4 (36.4%)	3 (27.3%)	7 (63.6%)	3 (2-4)
How effective do you think this activity is in improving your ability to perform your daily usual clinic activities?	0	1 (9.1%)	2 (18.2)	6 (54.5%)	2 (18.2)	8 (72.7%)	3 (2-3)
**Appropriateness**	Not acceptable at all	Somewhat acceptable	Acceptable	Very acceptable	Very much acceptable		
How acceptable / logical does this activity seem to you?				5 (50%)*	5 (50%)*	10 (100%)*	3.5 (3-4)
	Not suitable at all	Somewhat suitable	Suitable	Very suitable	Very much suitable		
How suitable/appropriate is this activity for the pediatric cardiology ambulatory clinic setting?	0	0	0	5 (45.5%)	6 (54.5%)	11 (100%)	4 (3-4)
**Risk**	Not severe at all	Somewhat severe	Severe	Very severe	Very much severe		
In your opinion, how severe (bad) are the risks of this activity (such as increased stress on staff or patients and families)?	11 (100%)	0	0	0	0	NA	0
**Suitability**	Not easy at all	Somewhat easy	Easy	Very easy	Very much easy		
How easy is it to apply this activity in the pediatric cardiology ambulatory clinic setting?	0	0	3 (27.3%)	4 (36.4%)	4 (36.4%)	8 (72.7%)	3 (2-4)
**Willingness to adhere**	Not willing at all	Somewhat willing	Willing	Very willing	Very much willing		
How willing are you to utilize this activity on an ongoing basis?	0		1 (9.1%)	4 (36.4%)	6 (54.5%)	10 (91%)	4 (3-4)

Abbreviations: IQR, interquartile range.

Scale: 0 = not at all; 4 = very much^12^.

* = one response missing

Two staff members provided responses to the open-ended question, “Is there anything else you want to tell us?”.

• Staff member #1: “Not sure how effective it will be in preparing teenagers for transfer but is an excellent tool.”
• Staff member #2: “Young adolescents are very difficult to reach in terms of planning ahead. It would be great to design an incentive.”

## 4 Discussion

In this study, we tested the effectiveness of a MA-facilitated transition activity in increasing discussion about transition, as well as staff acceptability of the intervention, in the pediatric cardiac ambulatory care setting. This intervention significantly increased the number of documented transition topics as compared with the number of topics documented in a similar cohort two years prior. We found that the transition activity led to documentation of discussion of a greater number of transition-related topics during the cardiology clinic visit compared with the number of topics that patients requested. Overall, staff appeared to accept the transition activity.

The significant increase in documented transition discussions with this MA-facilitated transition activity has important clinical implications. Studies have found that transition discussions are possibly more effective when embedded into routine pediatric cardiology care,^13^ and taking the time to address these topics on a routine basis is a critical component of preparing patients with CHD for a successful transfer to an adult model of care.^13^ We also found that more transition topics were documented than requested, indicating that this transition activity may have opened up the conversation as patients navigate their transition journey.^13^ Similar to others, we found that patients prioritized asking questions about medical transition topics in our study, such as understanding their heart defects and cardiac surgeries, the names of medications and what they are used for, and future needs for cardiology visits, among others.^6^

As mentioned previously, staff appeared to accept the transition activity, and staff indicated interest in continuing this activity on an ongoing basis. Interestingly, staff scored the effectiveness of transition activities in preparing pediatric patients with CHD for transfer to adult care and its effectiveness in improving their ability to perform their usual daily clinical activities lower than other items. However, the interpretation of what comprises ‘usual daily clinical activities’ could differ from staff member to staff member, based on staff roles and individual workflows.

MAs have served as health coaches in the primary care setting after undergoing 40 h of specialized training.^9^ No specialized training was required in this study other than what was presented at the pediatric cardiology division meeting. Additionally, MAs were provided with a prompt to introduce the study to patients. A known barrier to transition program implementation in CHD is a lack of additional staff to perform transition activities and clinic space.^1^ This MA-facilitated activity may be an amenable option to improving transition discussions. We believe that the activity was simple, effective and easy to implement, and the study did not require additional clinic space. Having time to perform transition activities is also a known barrier to transition program implementation,^1^ although we did not collect data on the time taken to perform this activity.

Despite the encouraging findings from this study, there are several limitations to consider. First, this study was conducted at a single center and over a short time frame. Second, this study did not collect patient-reported outcomes. Third, there was the possibility that study outcomes were impacted by the Hawthorne effect given that participants were aware that the study was taking place and may have influenced their behaviors to accommodate the study. Finally, the control group data was gathered from retrospective review of visits occurring two years prior. We acknowledge that there may be institutional and professional changes in awareness and of transition practices over that period. This study methodology also relies on the accuracy of historical documentation of transition conversations, but the comprehensiveness of that documentation cannot be ascertained retrospectively. Conversations may have taken place that were simply not documented which would thereby overestimate the treatment effect. Future research should include patient transition focused outcomes, including transfer success, measures of readiness for transition, and how this activity affects patients and families. Additionally, future research should include patient (and family) perceptions of the acceptability in the activity as convergent views are more likely to support successful implementation. The role of the MA in facilitating patient education, engagement and motivation is an area that is understudied and would benefit from further research.

## 5 Conclusion

Our study highlights that having an MA-facilitated transition activity increased documentation of transition discussions in the pediatric cardiology clinic setting. Staff were accepting of and in favor of continuing this low resource transition activity. More research is needed to better understand this intervention’s impact on patients with CHD’s long-term transition outcomes.

## Ethics approval

This study involving human participants was in accordance with the ethical standards of the institutional and national research committee and with the 1964 Helsinki Declaration and its later amendments or comparable ethical standards. The Human Investigation Committee (IRB) of Oregon Health and Science University approved this study. Staff completion of the questionnaire implied informed consent.

## Funding

This research did not receive any specific grant from funding agencies in the public, commercial, or not-for-profit sectors.

## CRediT authorship contribution statement

**Debora Burger**: Writing – review & editing, Writing – original draft, Methodology, Investigation, Formal analysis, Conceptualization, **Quin E. Denfeld**: Writing – review & editing, Writing – original draft, Supervision, Methodology, Formal analysis, Conceptualization, **Karen Uzark**: Writing – review & editing, Conceptualization, **Patrick D. Evers**: Writing – review & editing, Supervision, Methodology, Formal analysis, Conceptualization, **Andrew W. McHill**: Writing – review & editing, Supervision, Methodology, Formal analysis, Conceptualization, **Pam Ward**: Writing – review & editing, Data curation, **Reem Hasan**: Writing – review & editing, Supervision, Methodology, Formal analysis, Conceptualizatio.

## Declaration of Competing Interest

The authors declare that they have no known competing financial interests or personal relationships that could have appeared to influence the work reported in this paper.

## Data Availability

The authors do not have permission to share data.
